# Assessing the Informational Value of Large Language Models Responses in Aesthetic Surgery: A Comparative Analysis with Expert Opinions

**DOI:** 10.1007/s00266-024-04613-x

**Published:** 2025-02-18

**Authors:** Francesca Romana Grippaudo, Matteo Jeri, Michele Pezzella, Mariagiulia Orlando, Diego Ribuffo

**Affiliations:** Plastic Surgery Department, Sapienza University, Policlinico Umberto 1, Via Redi 2, San Giuliano Terme, Pisa, Rome, Italy

**Keywords:** Blepharoplasty, Dermal filler, Botulinum injection, A.I., ChatGpt, Google Bard

## Abstract

**Background:**

The increasing popularity of Large Language Models (LLMs) in various healthcare settings has raised questions about their ability to provide accurate and reliable information. This study aimed to evaluate the informational value of Large Language Models responses in aesthetic plastic surgery by comparing them with the opinions of experienced surgeons.

**Methods:**

Thirty patients undergoing three common aesthetic procedures—dermal fillers, botulinum toxin injections, and aesthetic blepharoplasty—were selected. The most frequently asked questions by these patients were recorded and submitted to ChatGpt 3.5 and Google Bard v.1.53. The answers provided by the Large Language Models were then evaluated by 13 experienced aesthetic plastic surgeons on a Likert scale for accessibility, accuracy, and overall usefulness.

**Results:**

The overall ratings of the chatbot responses were moderate, with surgeons generally finding them to be accurate and clear. However, the lack of transparency regarding the sources of the information provided by the LLMs made it impossible to fully evaluate their credibility.

**Conclusions:**

While chatbots have the potential to provide patients with convenient access to information about aesthetic plastic surgery, their current limitations in terms of transparency and comprehensiveness warrant caution in their use as a primary source of information. Further research is needed to develop more robust and reliable LLMs for healthcare applications.

**Level of Evidence I:**

This journal requires that authors assign a level of evidence to each article. For a full description of these Evidence-Based Medicine ratings, please refer to the Table of Contents or the online Instructions to Authors www.springer.com/00266.

## Introduction

Aesthetic plastic surgery has witnessed a significant rise in popularity in recent years. This trend has been driven by various factors, including increased media attention, changing beauty ideals, and advancements in surgical techniques [1]. As a result, patients are increasingly seeking information about aesthetic procedures to make informed decisions about their treatment options [2]. The three most popular aesthetic procedures in 2022 in USA according to the annual report of the American society of plastic surgery were: aesthetic blepharoplasty, botulinumum toxin injections, and dermal filler injections [3].

Blepharoplasty, also known as eyelid surgery, is a cosmetic procedure that aims to improve the appearance of the eyelids. It can address concerns such as sagging skin, wrinkles, and puffy bags, resulting in a more youthful and rejuvenated look. Recovery time is relatively short, with most patients able to resume their normal activities within a week. Blepharoplasty can be an effective way to enhance the appearance of the eyes and create a more harmonious facial balance [4].

Botulinum toxin injections, commonly known as Botox injections, are a minimally invasive cosmetic procedure that utilizes botulinum toxin type A to temporarily relax facial muscles. This relaxation reduces the appearance of wrinkles, particularly dynamic wrinkles, which are formed during facial expressions such as frowning, squinting, and smiling. The effects of botulinum toxin injections typically last for 3-4 months, after which the muscles gradually regain their function [5].

Dermal filler injections, also known as soft tissue augmentation, are a minimally invasive cosmetic procedure that involves injecting gel-like substances into the skin to restore volume, smooth wrinkles, and enhance facial contours. The effects of dermal filler injections typically last for 6-12 months, depending on the type of filler used and the treatment area [6].

To patients preferring a non-surgical approach, botulinum toxin injections and dermal filler can be offered. This alternative that can be performed in an in-office setting does not require the use of local anesthetics and does not produce scars; however, the result is not permanent [5–7].

In this context, Large Language Models (LLMs) have emerged as a potential tool for providing patients with accessible and convenient access to information about aesthetic plastic surgery [1, 8]. A Large Language Model processes and generates human-like text across diverse tasks and topics and can simulate conversation with humans, and they have been successfully implemented in various industries, including customer service and healthcare [9].

However, the use of LLMs in aesthetic plastic surgery raises concerns about the accuracy and reliability of the information they provide: the potential risks associated with aesthetic procedures highlight the need of ensuring that patients must have access to accurate and up-to-date information to make informed decisions and to have the right expectations.

Against this backdrop, this study aimed to verify the informational value of LLMs responses in aesthetic plastic surgery by letting experienced surgeons evaluate them.

## Methods

### Patient Selection

For each of these three common aesthetic procedures—dermal fillers, botulinum toxin injections, and blepharoplasty—we recruited ten patients from different plastic surgery clinics. These procedures were selected due to their high popularity [3] and the availability of experienced surgeons performing them.

### Question Collection

During the pre-operative consultation, patients were asked to list three questions regarding their chosen procedure. These questions were then categorized into themes based on their content. We focused our research on three recurring themes for each procedure. For each theme, we generated a question that was translated from Italian into English.

The three more commonly asked questions regarding dermal fillers were: “Is the injection of dermal fillers painful?”, “How often will I need to have dermal fillers?”, “How long does the effect of dermal fillers last?”.

The three more commonly asked questions regarding botulinum toxin injections were: “Is the injection of botulinum toxin painful?”, “How often will I need to have botulinum toxin injections?”, “How long does the effect of botulinum toxin injections last?”.

The three more commonly asked questions regarding aesthetic blepharoplasty were: “Is the aesthetic blepharoplasty painful?”, “Will I have visible scars after an aesthetic blepharoplasty?”, “How long does the results of an aesthetic blepharoplasty last?”.

### LLMs Responses

**Fig. 1:**
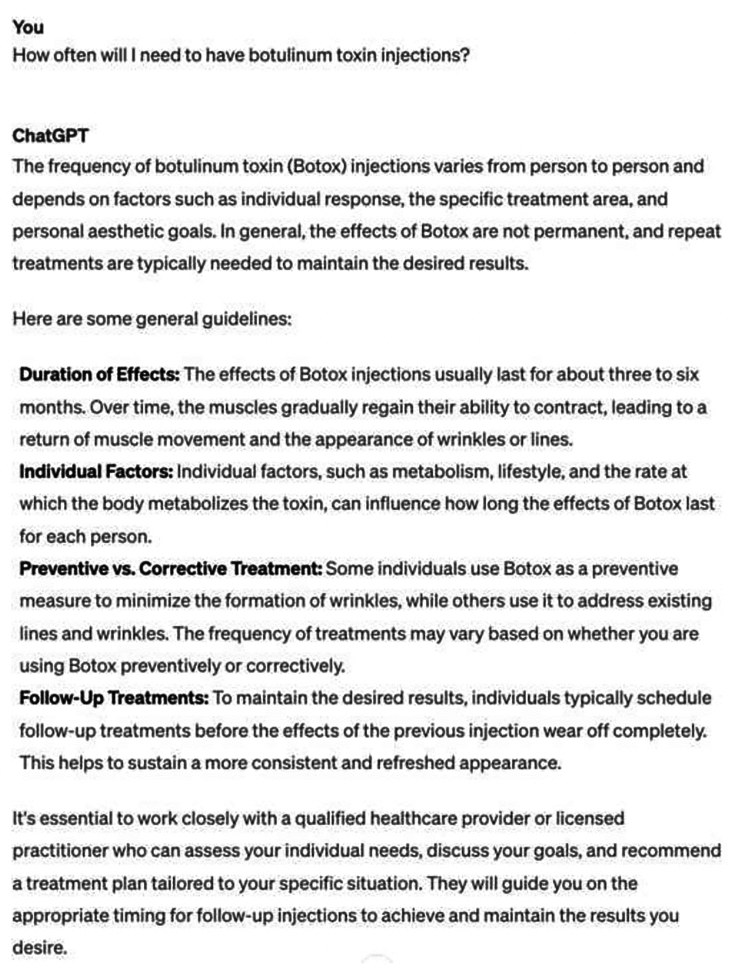
Picture illustrating an example of answer given by the large language model ChatGpt regarding the frequency of botulinum toxin injections

**Fig. 2:**
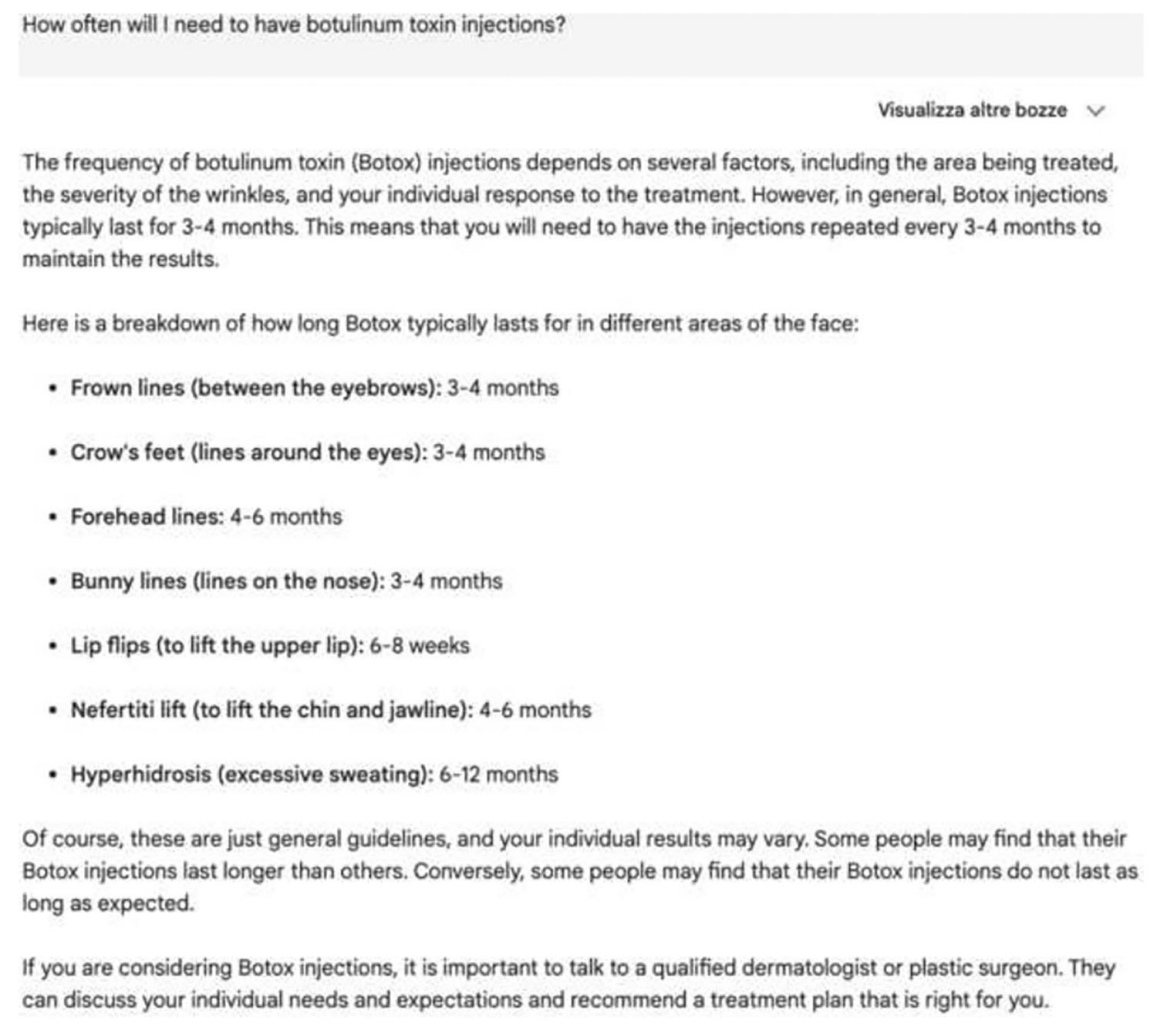
Picture illustrating an example of answer given by the large language model Google Gemini regarding the frequency of botulinum toxin injections

The categorized questions were submitted to ChatGpt 3.5 and Google Bard v1.53, two popular LLMs platforms [10], simulating a conversation between a patient and the doctor performing the procedure. The inquiry was performed on the same date for both the LLMs from the same account specifically created for the occasion. The chatbots were instructed to provide comprehensive and informative answers to each question. Every first answer was recorded, not including in our study misinterpretations of the questions from the LLMs or corrections later provided. In Figs. 1 and 2 is listed an example of the answers given, respectively, by ChatGpt and Google Bard to the question: “How often will I need to have botulinum toxin injections?”.

**Fig. 3:**
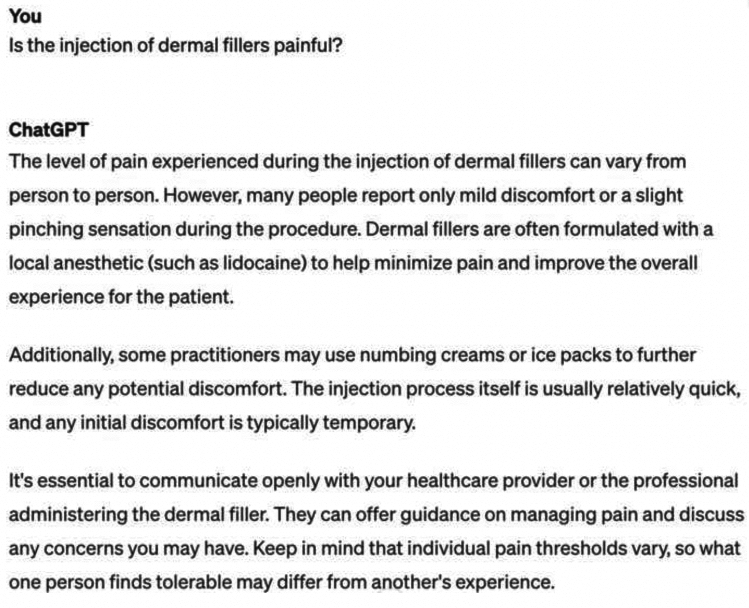
Picture illustrating an example of answer given by the large language model ChatGpt regarding the painfulness of dermal fillers injections

**Fig. 4:**
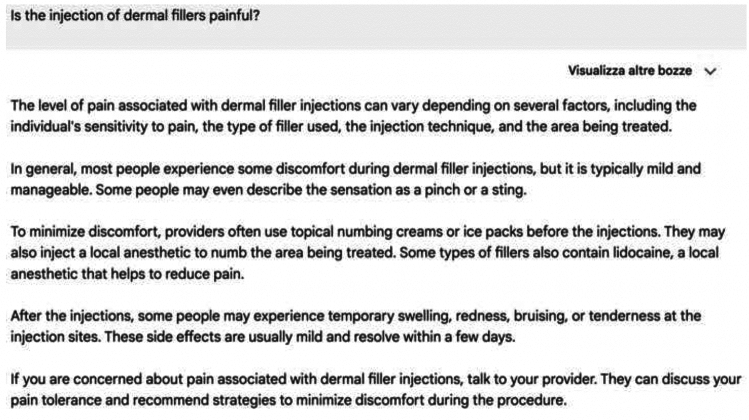
Picture illustrating an example of answer given by the large language model Google Gemini regarding the painfulness of dermal fillers injections

In Figs. 3 and 4 is listed an example of the answers given, respectively, by ChatGpt and Google Bard to the question: “Is the injection of dermal fillers painful?”.

### Expert Evaluation

Thirteen experienced aesthetic plastic surgeons were recruited to evaluate the chatbot responses. The surgeons were provided with the questions and the corresponding LLMs responses, and they were asked to rate the answers on a Likert scale for accessibility, accuracy, and overall usefulness [11], with scores from 1 (completely disagree with answer) to 5 (completely agree with answer).

### Data Analysis

The ratings from the surgeons were analyzed using descriptive statistics to assess the overall quality of the LLMs responses. Every surgeon was given an answer sheet where to express its rating. The answer sheets were than collected and the scores from 1 to 5 given to each aspect required to evaluate about the answers generated by the LLMs. For every answer were calculated the mean value, the median value, and the lower and upper interquartile range and they are reported in three tabs.

## Results

### Chatbot Response Quality

The responses were generally found to be of good quality; 40 answers out of 48 had a mean value of the scores given by the surgeons greater than 4 and for all the answers the mean value was greater than 3.4615. The overall mean value of the scores all the answers by the surgeons was 4.2906, median value 4 (IQR 4-5). Our results do not differ from the data in the literature [12].

For the aesthetic blepharoplasty answers, the mean value of the scores given by the surgeons was 4.235, median value 4 (IQR 4-5). For this topic the answers given by Google Bard had lower scores than the ones given by ChatGpt, the mean value of the scores given to Google Bard’s answers was 4.0170, median value 4 (IQR 4-5), the mean value of the scores given to ChatGpt’s answers was 4.4530, median value 4 (IQR 4-5).

For the botulinumum toxin injections answers the mean value of the scores given by the surgeons was 4.3290, median value 4 (IQR 4-5). Also, for this topic the answers given by Google Bard had lower scores than the ones given by ChatGpt; the mean value of the scores given to Google Bard’s answers was 4.1111, median value 4 (IQR 4-5); the mean value of the scores given to ChatGpt’s answers was 4.5470, median value 4 (IQR 4-5).

For the dermal fillers answers, the mean value of the scores given by the surgeons was 4.3077, median value 4 (IQR 4-5). In this case, the answers given by Google Bard gained higher scores than to ones given by ChatGpt; the mean value of the scores given to Google Bard’s answers was 4.4615, median value 4 (IQR 4-5); the mean value of the scores given to ChatGpt’s answers was 4.1538, median value 4 (IQR 4-5).

## Discussion

According to the plastic surgeons interviewed, the quality of the answers given by ChatGpt was higher than Google Bard resulting in more accurate, more useful, and more accessible information when searching for aesthetic blepharoplasty and the botulinum toxin injections. Differently when asking about dermal fillers injections, Google Bard gave better answers according to our analysis.

### Source of Information

The chatbots were not able to provide the sources for their responses. This lack of transparency made it impossible to fully evaluate the credibility of the information provided [13].

The findings of this study suggest that chatbots have the potential to provide patients with convenient access to information about aesthetic plastic surgery. The chatbot responses were generally found to be accurate and clear, and they were perceived as being useful by experienced surgeons.

However, the lack of transparency regarding the sources of the information provided by the chatbots raises concerns about their credibility [13]. Without knowing the sources, it is impossible to assess the validity and reliability of the information. ChatGpt 3.5 has a limited dataset which was updated for the last time in January 2022 [14], but Google Bard’s dataset is also based on Google search and potentially all the fully accessible information on internet are accessible from Google Bard’s algorithm [15]. This is a significant limitation, as aesthetic plastic surgery is a complex field with potential risks, and it is crucial for patients to have access to accurate and trustworthy information to make informed decisions about their treatment options. Both LLMs do not discriminate between scientifically proved information and unverified ones. Both LLMs, in every question asked, realize the medical nature of the question and at some point of the answer, in general at the very beginning, suggest the user to call on a certified physician and not on only rely to the answers given.

While [16] it can be objected that neither a practicing plastic surgeon when inquired about the source of the information given to a patient might be able to cite each and every paper or text she/he bases her/his knowledge on, in every doctor–patient interaction in the outpatients setting we assume a certain degree of authority principle,

The authority principle in medicine refers to the phenomenon where individuals tend to trust and follow the recommendations, of those perceived to have expertise, experience, or official status in the medical field. Medical professionals undergo extensive education and training, which endows them with specialized knowledge and skills. Years of clinical practice and exposure to a wide variety of cases build a repertoire of knowledge that is often not captured fully by textbooks or initial training.

If given the time, a doctor can almost always produce the sources to back up her/his claims.

Every kind of information contained or processed through an LLM is not validated or even overviewed from any kind of scientific society or institution. The responses given by LLMs are the byproduct of an algorithm developed by private companies, and neither the source of information or the information retrieval process can be analyzed. Moreover, the people who developed the algorithms behind LLMs are not plastic surgeons or even practitioners at all.

## Conclusion

Chatbots offer a promising avenue for providing patients with accessible information about aesthetic plastic surgery. However, further research is needed to address the limitations of current chatbots [17], particularly in terms of transparency and comprehensiveness. In the meantime, caution should be exercised in using chatbots as a primary source of information for aesthetic plastic surgery, and patients should always be encouraged to consult with a physician.
