# Correction: Assessing the Informational Value of Large Language Models Responses in Aesthetic Surgery: A Comparative Analysis with Expert Opinions

**DOI:** 10.1007/s00266-025-04789-w

**Published:** 2025-03-11

**Authors:** Francesca Romana Grippaudo, Matteo Jeri, Michele Pezzella, Mariagiulia Orlando, Diego Ribuffo

**Affiliations:** Plastic Surgery Department, Sapienza University, Policlinico Umberto 1, Via Redi 2, San Giuliano Terme, Pisa, Rome, Italy

**Correction to: Aesthetic Plastic Surgery** 10.1007/s00266-024-04613-x

The original online version of this article was revised to correct the presentation of the name of coauthor Mariagiulia Orlando.

The original article has been corrected.

